# Ravens (*Corvus corax*) use conspecifics and heterospecifics to navigate risky foraging scenarios

**DOI:** 10.1093/beheco/arag093

**Published:** 2026-07-30

**Authors:** Silvia Damini, Arnout Lindeman, Awani Bapat, Petra Sumasgutner, Thomas Bugnyar

**Affiliations:** Department of Behavioral and Cognitive Biology, Faculty of Life Sciences, University of Vienna, Djerassiplatz 1, Vienna 1030, Austria; Konrad Lorenz Research Center for Behavior and Cognition, Core Facility of the University of Vienna, Fischerau 13, Grünau im Almtal 4645, Austria; Behavioural Ecology Group, Animal Sciences Department, Wageningen University and Research, Droevendaalsesteeg 4, Wageningen 6708, The Netherlands; Behavioural Ecology and Sociobiology Unit, German Primate Center, Leibniz Institute for Primatology, Kellnerweg 4, Göttingen 37077, Germany; Department of Behavioral and Cognitive Biology, Faculty of Life Sciences, University of Vienna, Djerassiplatz 1, Vienna 1030, Austria; Konrad Lorenz Research Center for Behavior and Cognition, Core Facility of the University of Vienna, Fischerau 13, Grünau im Almtal 4645, Austria; Department of Behavioral and Cognitive Biology, Faculty of Life Sciences, University of Vienna, Djerassiplatz 1, Vienna 1030, Austria; Konrad Lorenz Research Center for Behavior and Cognition, Core Facility of the University of Vienna, Fischerau 13, Grünau im Almtal 4645, Austria; Department of Behavioral and Cognitive Biology, Faculty of Life Sciences, University of Vienna, Djerassiplatz 1, Vienna 1030, Austria; Konrad Lorenz Research Center for Behavior and Cognition, Core Facility of the University of Vienna, Fischerau 13, Grünau im Almtal 4645, Austria

**Keywords:** scavengers, scrounging, producer-scrounger game, kleptoparasitism, exploration test

## Abstract

Foraging animals need to balance the trade-off between the benefits of exploiting new resources and the unknown risks they entail. Changing environments are especially challenging, as this balance between risk and reward needs to be constantly reevaluated. One strategy animals, and in particular scavengers employ, is using conspecifics and heterospecifics as sources of information to access food. In this study, we focused on free-ranging ravens that habitually forage in a wildlife park together with heterospecifics (boars) and tested their responses to unfamiliar and unpredictable foraging situations. In the 2-sites experiment (E1), we presented them with a choice between an unfamiliar (new) and a familiar (old) foraging site; in the object experiment (E2), we presented them with an object, otherwise not seen in the wildlife park, positioned randomly next to 1 of the sites. In both experiments, we found that ravens delayed their initial approach until the boars reached the food. In E1, ravens took longer to approach the food; stole more food from conspecifics but visited in larger numbers at the new site compared with the old. In conclusion: they flexibly used others, including heterospecifics, as sources of knowledge and means to access food. In E2, the object had a stronger aversive effect at the old site than at the new site, suggesting that the effect of unpredictability is stronger in familiar environments, where it might cause an expectancy violation. Our findings highlight that the behavior toward conspecifics and heterospecifics should be considered when studying scavengers' adaptability to changing environments.

## Teaser

Ravens use wild boars as “first tasters” to assess the safety of a foraging site. Unfamiliar and unpredictable situations can be risky for scavengers. Ravens can reduce this uncertainty by waiting for other species to forage and confirm the safety of a site, by foraging in larger groups, and by stealing food from other ravens instead of approaching an unknown site themselves. These tactics could make ravens, and possibly other scavengers, more adaptable to environmental changes.

## Introduction

Foraging animals need to balance the trade-off between the benefits of exploiting new resources and the risk of being exposed to unfamiliar, unpredictable, and therefore potentially dangerous stimuli (Kacelnik and Bateson 1997). Yet, individuals often show substantial variation in risk taking behavior between and within species (Fodrie et al. 2012; Monadjem et al. 2018; Gonzálvez et al. 2021; Olson et al. 2022). Part of this variation can be explained by social and/or environmental factors (eg Sih 2013); some of this variation tends to be consistent within individuals and may reflect their personality or temperament (Réale et al. 2007). In either case, the individuals' behavioral responses to perceived risk can have huge implications on their survival and fitness (Tuomainen and Candolin 2011; De Guinea et al. 2026).

Optimizing risk taking behavior is particularly relevant in rapidly changing environments, where the balance between risk and reward needs to be constantly reevaluated (Sih et al. 2011; Lowry et al. 2013; Gaynor et al. 2018). Changing environments may contain aspects of novelty and give rise to 2 opposing behavioral traits: neophobia and neophilia, respectively fear of and attraction to the new (eg Brown et al. 2013). On 1 hand, neophobic reactions benefit individuals by reducing their engagement with potentially dangerous stimuli (Brown et al. 2013, 2015; Crane et al. 2020), but they also reduce the time individuals spend exploring and claiming new resources (Lima and Dill 1990; Crane et al. 2020; Szabo and Ringler 2023). Neophilic behaviors, on the other hand, can be beneficial by facilitating information gathering in novel environments, but they have the potential of putting the animal in risky situations (Souganidis et al. 2024). Note that for a stimulus to be considered novel, it should be completely unknown to the study subjects; however, this is rarely the case in nature, as most stimuli occur in versions that share (some) characteristics and/or are encountered multiple times, sometimes across different contexts. Therefore, in changing environments animals tend to experience degrees of unfamiliarity, in the form of lesser-known objects, foraging opportunities, dangerous heterospecifics, etc. (Crane et al. 2024). For scavengers in particular, such unfamiliarity is often embedded in a broader ecological network, because environmental change can affect not only the resource itself, but also which species discover it first and how access to it is socially mediated (Marie Montenegro et al. 2025). Thus, changing environments may modify both the perceived risk of a resource and the social pathways through which information about that resource becomes available.

Since unfamiliarity denotes lack of knowledge about the characteristic of a certain stimulus or environment (McNamara and Houston 1987; Blumstein 2006; Réale et al. 2007), it also entails its unpredictability. Predictability is the measure of consistency an individual experiences in an environment, allowing it to understand and adjust to the spatiotemporal consistency of stimuli (Stephens 1993; Riotte-Lambert and Matthiopoulos 2020; Vardi and Berger-Tal 2022). By contrast, animals in environments with low or no predictability (hereafter referred to as “unpredictable”), experience a reduced possibility to estimate and learn about risks (Crane et al. 2024). Accordingly, prey animals, such as northern red-bellied dace (*Chrosomus eos*), respond more aversively to temporally random predation risk compared with temporally consistent risk (Brown et al. 2020). Similarly, Trinidadian guppies (*Poecilia reticulata*) remain averse to unfamiliar odors that are presented in a spatially unpredictable manner, whereas they do not remain averse when the odors are spatially consistent (Feyten et al. 2019). Taken together, unpredictability and unfamiliarity increase the perception of uncertainty in the environment and therefore, they entail risk (Crane et al. 2020, 2024).

A powerful strategy to diminish the risk in accessing unpredictable/unfamiliar and thus potentially dangerous resources is to gather information by observing conspecifics and heterospecifics interacting with them (eg Hromada et al. 2008; Greggor et al. 2016)). The extensive literature on social learning indicates that a variety of species pay attention to conspecifics when it comes to foraging or anti-predator decisions in unfamiliar or uncertain environments (Hoppitt and Laland 2013). For example, migrant orangutan males observe conspecifics to gain ecological knowledge about new environments (Mörchen et al. 2023), while jackdaws (*Corvus monedula*) use information from conspecifics to assess the risk of novel food items (Greggor et al. 2016). Like with conspecifics, information from heterospecifics is frequently used across taxa in the context of locating food, territory and nesting site selection. For example, collared flycatchers (*Ficedula albicollis*) utilize song quality information from great tits (*Parus major*) to select suitable nesting sites (Morinay et al. 2020a, 2020b). Bumblebees (*Bombus terrestris*) are able to use cues provided by heterospecifics (honeybees, *Apis mellifera*) to locate foraging opportunities (Dawson and Chittka 2012). Furthermore, coyotes (*Canis latrans*) and different species of raptors such as Golden eagle (*Aquila chrysaetos*), Bald eagle (*Haliaeetus leucocephalus*), and Griffon vulture (*Gyps fulvus*) use corvids to access carcasses (Orr et al. 2019), often exploiting their early detection abilities.

Interspecific information use can shape how animals evaluate both opportunity and danger at shared resource sites. This perspective is especially relevant for scavengers, whose access to ephemeral resources often depends on tracking the behavior of other species at carcasses or feeding sites (Tóth et al. 2020; Marie Montenegro et al. 2025; Loretto et al. 2026). Amongst them, are many corvid species that show behavioral traits associated with foraging under risk, like pronounced neophobia (Miller et al. 2022) and high behavioral plasticity in exploiting the knowledge and skills of others (Loretto et al. 2026). Common ravens (*Corvus corax*), for example, develop neophobia around the time of dispersal (Miller et al. 2015) but remain plastic in how strongly they show fear responses to novel stimuli (eg depending on social constellations such as the number of, and social relationship to, foraging partners Heinrich and Marzluff 1991; Stöwe et al. 2006a, 2006b; Stöwe and Kotrschal 2007). Older ravens also adjust their foraging strategies depending on perceived risk, as they time their departure with food in order to avoid conspecific kleptoparasitism (Gallego-Abenza et al. 2020). Moreover, foraging ravens may use alarm calls of other corvids as a source of information about predation risk (Davídková et al. 2020) and mammals such as wolves (*Canis lupus*) as means to gain access to food (Ho et al. 2023). The latter may take a similar form as with conspecifics, ie stealing the food from the animal; alternatively, they may manipulate the animals by pecking or pulling their fur to make them move away from food (Bugnyar and Kotrschal 2002). In either case, the ravens' behavior could be interpreted as scrounging (Barnard and Sibly 1981), as they exploit resources that have been made available by others. Recent evidence further indicates that ravens use large-scale spatial memory to revisit areas with recurrent wolf kills, suggesting that personal information, memory, and social information may jointly support scavenging decisions across broad spatial scales (Loretto et al. 2026). Likewise, work on another apex avian scavenger, the wedge-tailed eagle (*Aquila audax*), shows that carrion use and competitive social interactions vary predictably with habitat openness, season, and carrion value (Meisuria et al. 2024), reinforcing the idea that scavenging behavior is tightly linked to both ecological context and social competition. What is still missing, however, are experimental approaches, testing the ravens' use of con- and heterospecifics when confronted with specific characteristics of changing environments, such as unfamiliarity and unpredictability, in the foraging context.

We here investigated the behavior of free-flying ravens scrounging food at daily feedings of captive wild boars (*Sus scrofa*) in a local zoo in the Austrian Alps. In 2 consecutive experiments, we manipulated aspects of the foraging environment stepwise, exposing ravens to scenarios with variable levels of familiarity and predictability. In the 2-sites experiment, we introduced an additional feeding site (new site), where we provided the same food quality and quantity as in the original (old) feeding site, twice per week for a month. We hypothesized that ravens would initially perceive the new site as riskier, due to its unfamiliarity and unpredictability, but that over time they would become familiar with the procedure and eventually treat it similarly to the old site. This behavioral adjustment should be reflected in changes over time, for example a decrease in kleptoparasitism, and therefore a decrease in agonistic behavior as they can be strongly correlated (Heinrich and Marzluff 1991). Accordingly, we predicted that ravens would be slower to approach the new site initially relying on behavioral cues from heterospecifics, and that they would modify their behavior toward conspecifics and heterospecifics. Specifically, we expected ravens (i) to rely on the wild boars to initiate feeding at the new site, (ii) to show more kleptoparasitism attempts toward conspecifics and (manipulation of) heterospecifics at the new site, and (iii) to engage in more agonistic interactions at the new site compared with the old site.

In the object experiment, we presented an object (bicycle) next to 1 of the 2 feeding sites. Note that the ravens foraging in the park are likely used to bikes; yet, they have never encountered a bike in the context of a wild boar feeding before. Furthermore, we randomized the bike's position (at old or new site), making the scenario unpredictable throughout the experiment. We hypothesized that the ravens would perceive the site next to the bike as risky. Accordingly, we predicted that ravens (i) might rely on the boars to initiate feeding at the site with the object, (ii) show more kleptoparasitism attempts toward conspecifics and manipulation of heterospecifics, and (iii) engage in more agonistic interactions at the site with the object across the experiment. However, we did not expect any consistent difference between the old and the new site in the object experiment.

## Materials and methods

### Study system

The experiments were conducted in Austria at the Cumberland Wildpark (47°48′25″N 13°57′00″E). In this area, wild ravens take advantage of the resources of the park by scrounging food from various animal enclosures. This population has been the subject of various studies since 2008 and forms mixed-sex groups composed of all age classes but mainly non-breeders. Non-breeder groups are highly dynamic, changing in size and composition within the day and across the annual cycle (Braun and Bugnyar 2012; Loretto et al. 2017). On average, around 50 ravens participate at daily feedings, whereby about half of the birds can be individually identified by their rings and wing tags (see Table S1). While most of these ravens originate from the wild, around 20% of the marked birds are offspring from our captive colony (ie they were raised by their parents in aviaries and then allowed into free flight, eventually integrating into the wild flock).

### Experimental area

We conducted the experiments in a wild boar enclosure (*Sus scrofa*), consisting of a field with sloped sides overlaid by trees (approximate area: 8.000 m^2^). Specifically, the experiments were carried out in the areas where the boars are fed each morning and where the ravens have been regularly observed scrounging from con- and heterospecifics (see Image D1 supplementary material). During our experiments, 5 adult boars and between 10 and 12 piglets were held in the enclosure and were present during each test. Only the adults were included in our analysis. While the piglets were present at most feeding events, they were not yet manipulating food themselves and thus were largely ignored by the ravens. The boars do not pose an imminent threat to the ravens (ie we do not have any records of ravens being actively killed by boars). On the contrary, the boars are often pecked, ridden, or in other ways manipulated by ravens to better access food. Routine observations of the ravens have been done in this area year-round for the past 18 yr, always following the same protocol: researchers provide the food to the boars at the same location inside the foraging area, then they move to the observation post situated 15 m away from the feeding site. The ravens and boars are habituated to the researchers' presence, and this foraging scenario is familiar to them. Furthermore, this long-term standardized procedure renders it a highly predictable foraging site for the ravens.

### Experimental procedure

We conducted 2 consecutive experiments in which we manipulated the standard boar feeding setup in different ways.

**Figure 1:**
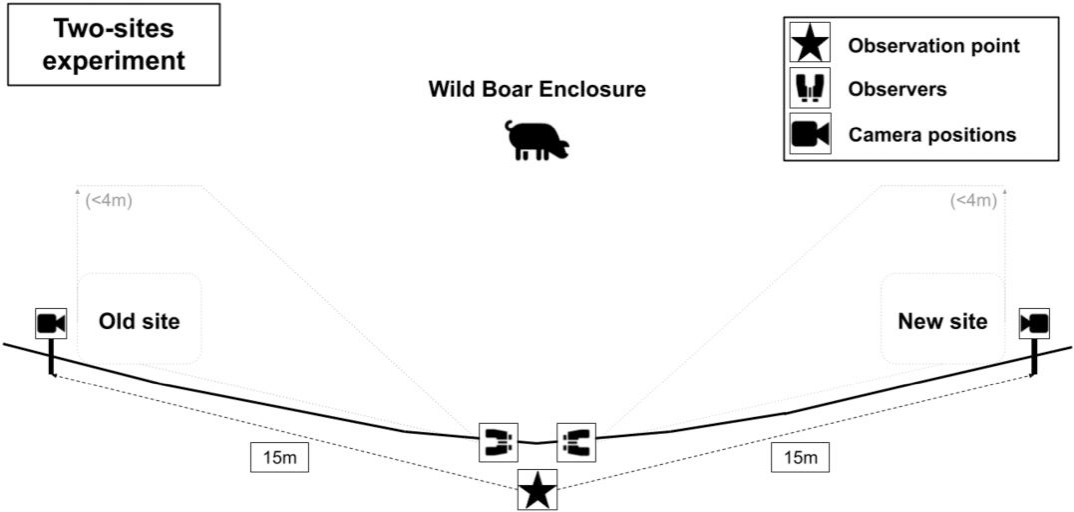
Setup for the 2-sites experiment. The 2 feeding sites are equidistant from the observation post (indicated by binoculars), whereby the old feeding site is on the left, the new feeding site is on the right (see camera positions).

The 2-sites experiment was conducted in spring (March–April) of 2 consecutive years (2022, 2023), during 5 wk each year for a total of 20 trials. The trials were always run in the morning between 8 and 10 AM and only 1 trial was run per day. During each trial we introduced a second feeding site (new site) to the right of the observation post, at 30 m distance to the regular feeding site (old site; see Fig. 1). The 2 sites were located within a line of sight, ie the area was free of plants or any other barriers, so that boars and ravens could see both sites at the same time. The new site was presented 2 times per week on a semi-random schedule (see Appendix S1 for complete schedule). The food was divided equally between sites and comprised a variable mixture of pellets, bread, meat, and fish. The food quality and quantity were equally familiar and predictable between the 2 sites. Both sites were filmed using GoPro cameras. The feeding procedure followed the standard protocol but was conducted by 2 researchers simultaneously: they carried the food to the 2 sites, set up the cameras and waited for 3 min before they simultaneously delivered the food into the enclosure and returned to the observation post. Each researcher focused on 1 of the sites and voice-recorded behavioral observations until the food at both sites was consumed by the boars and ravens. Due to the fission-fusion dynamics of raven groups, some individuals participated in the experiment in both years, others only in 1 yr (see Table S1 for detailed list).

In 2023, after repeating the 2-sites experiment, we additionally performed the object experiment for 4 wk for a total of 10 trials. The trials were always run in the morning between 8 and 10 AM and only 1 trials was run per day. This time, during each trial we always presented food at the 2 sites (old and new), but from time to time we added an object (bicycle) next to 1 or the other site to create an unpredictable environment. Overall, we had 3 conditions: 2 sites—without object, 2-sites—with object at the old site, 2-sites—with object at the new site, which we administered in a semi-randomized order (whereby the same condition could not occur more than twice in a row; see Appendix S1 for complete schedule). At the beginning of an object condition, the researchers placed the bike on the outside of the fence next to the designated feeding site and then followed the same procedure as described in the 2-sites experiment.

With the introduction of the new site in the 2-sites experiment, we simulated the appearance of a new resource, which initially constitutes an unfamiliar and unpredictable aspect in the environment for the animals. Yet, since the new feeding site is always at the same position, it should become spatially familiar and predictable; note that there was no contingency nor constancy in the time when the site was used, ie the new site remained temporarily unpredictable (compare Riotte-Lambert and Matthiopoulos 2020). On the contrary, in the object experiment the object was presented at random next to 1 of the feeding sites, so that it was not possible for the ravens to predict its presence, neither spatially nor temporally. We chose bicycles as objects, because they are familiar to the ravens, as they are frequently used by recreationists in the areas surrounding the park. In total, 1 out of 5 different bicycles were randomly chosen for each trial.

### Data collection

We utilized a combination of scan and ad libitum behavioral sampling to collect the data, both for the ravens and the wild boars' behaviors (Altmann 1974). All variables were sampled and coded separately for each site. We used the moment that a raven ate or grabbed food within half a meter of the food pile as the starting point of the data collection at that site. From that moment, we used synchronized video and audio observations to count specific behaviors and individual presence on a per-minute basis (see Table 1). Some of the behaviors were only added to the ethogram in 2023 (Table 1). During this minute, it was possible for both ravens and boars to move between sites, and for their behavior and presence to be recorded at both sites. If ravens ate at only 1 site during a trial, we used the same starting point for both sites. We measured the ravens' latency to approach from when the food was made available in the enclosure, to when at least 5 ravens had landed at the site. We decided on this threshold as given ravens' behavior and the limitations of our setting it would give us a more precise and conservative measure. The boars' latency to eat was measured from when at least 1 boar grabbed food or ate directly at the site (see Table 1). We considered ravens as present at the feeding site if they were on the ground, on low tree stumps, or on boars, and within 4 m of the food pile. We used the number of defensive calls produced by the ravens as a proxy for agonistic interactions. Data collection lasted for 15 min or until the food was finished.

**Table 1:** Summary of the variables coded with information on the year the data was collected (“Year data collection” column), whether they were coded from audio, video, or both (“Source” column), and description (“Description” column).

Variable	Year data collection	Source	Description
Ravens' latency to approach (s)	2022, 2023	Video	Time from when the food is made available to when at least 5 ravens land at the site.
Boars' latency to eat (s)	2022, 2023	Video	Time from when the food is made available to when a boar eats or grabs food from the site.
Number of ravens	2022, 2023	Video, audio	Number of ravens on the ground, on low tree stumps, or on boars, and within 4 m of the food pile, collected through 1-min scans. If ravens were at the edge of the 4 m, we considered them present at the site only if they were oriented toward it. The number of ravens used in the analysis was the product of the cumulative total across scans within a trial.
Number of kleptoparasitism attempts	2023	Video, audio	Number of stealing attempts between ravens, collected through 1-min scans.
Number of agonistic interactions	2023	Video, audio	Number of defensive calls per minute.
Number of boars	2022, 2023	Video	The number of adult boars within 1 m of the biggest food cluster, collected through 1-min scans.
Boar manipulation	2023	Video, audio	Number of times ravens are observed pecking, pulling fur, and/or riding the adult boars.

### Data analysis

We conducted our analysis in R (version 4.3.1; R Core Team (2023)), using a mix of linear models (LMs) and generalized LMs (GLM, Baayen 2007), depending on the data structure. For all analyses, we used an information theoretic approach and model averaging with the package MuMIn (Bartoń 2013). For each question, a set of candidate models was generated. All candidate model sets contained the global model with the variable “day” as predictor and all relevant covariates (list), the null model (“day” variable only), and a set of candidate models always including the key day status predictor term and all possible nested combinations of the fixed covariates appropriate to that model. Since we only had 1 trial per day, the data for each trial was independent and we only used the variable day as a fixed effect to account for the variance arising from possible factors specific to a testing day. The models within a set were then ranked using ΔAIC_c_ values (see Tables S2 to S11). Akaike weights (*ω_i_*) were calculated to assess the relative likelihood for each model considered (Burnham and Anderson 2004). Thus, *ω_i_* reflected model probability given the full model set rather than only those below a given threshold of ΔAIC_c_. A table of best candidate models was extracted, and if more than 1 model was under the ΔAIC_c_ = 2.0 threshold, model averaging was used (see Tables S10 and S11; Anderson et al. 2001). We report the direction of parameter estimates and their magnitudes (effect sizes), and adjusted SEs and CIs (95% confidence limit) from model-averaged coefficients. We based our model selection procedure on the principle of parsimony: the largest amount of variance explained with the minimum number of predictors (Burnham and Anderson 2004). For both experiments, the response variables were the ravens' latency to approach the site, the number of ravens present at the site, the number of kleptoparasitism attempts, the number of boar manipulations, and the number of defensive calls (proxy for agonistic interactions). For the 2-sites experiment, the main predictors were day, site position (old, new), and latency for the boars to approach the site. For the object experiment, the main predictors were day, site position (old, new), the presence of the object, and the interaction between the presence of the object and site position. To investigate the ravens' latency to approach, we included the latency for the boars to eat as a main predictor in both experiments.

All the log transformations were done using the natural logarithm (base e). The latency for a minimum of 5 ravens to approach was analyzed using the log-transformed variable in a LM. To investigate the number of ravens present at the foraging sites, we analyzed the number of ravens (cumulative total across scans within a trial, the identity of the individual ravens was not taken into account) in an LM. The total number of kleptoparasitism attempts, agonistic interactions, and boar manipulations were averaged across the number of ravens and then analyzed using LMs. For kleptoparasitism, agonistic interactions, and boar manipulations, only data from 2023 was used because of methodological differences with 2022. For all models, post hoc comparisons were done using the Tukey HSD test.

For the 2-sites experiment, it was not possible to determine the ravens' latency to approach for 1 trial because of a problem with the video recording. For the object experiment, it was not possible to count the amount of kleptoparasitism attempts, agonistic interactions, and boar manipulations for 1 trial because of problems with the audio recording. The total sample sizes for each variable tested were a result of the number of trials where data was collected for that specific variable multiplied by 2, as data was collected for both sites each time (sample sizes 2-sites experiment: raven's latency to approach = 38, number of ravens = 40, kleptoparasitism attempts = 20, agonistic interactions = 20, boar manipulations = 20; sample sizes object experiment: raven's latency to approach = 20, number of ravens = 20, kleptoparasitism attempts = 18, agonistic interactions = 18, boar manipulations = 18).

## Results

### Two-sites experiment

#### The latency to approach was between 16 and 1,427 s, with an average of 162 s.

**Figure 2:**
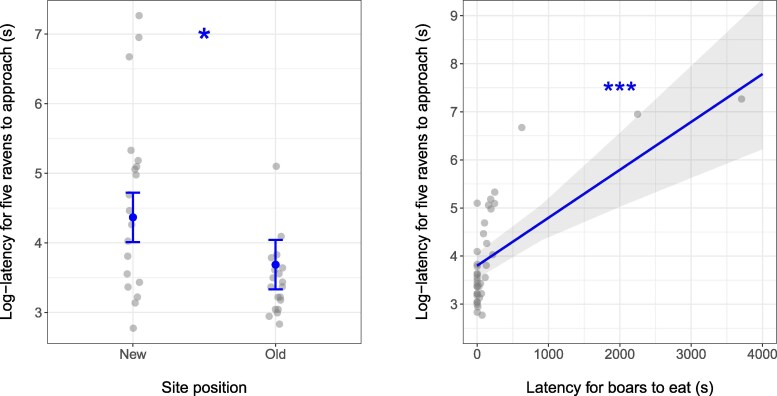
(left) Ravens approached the new site more slowly than the old site, and (right) their approach latency increased with the boars' feeding latency. Panels show, the effect of site and the latency for boars to eat (in seconds), on the log-latency for 5 ravens to approach during the 2-sites experiment. The dots represent individual data points. The error bars and the ribbon represent the lower and upper bounds (95% confidence interval) of our LM, with the dot on the error bars and the blue line as the mean model fit. Detailed effect sizes can be found in Table 2.

Test condition (old/new site) and boars' latency but not time (experimental days) had a significant effect on the ravens' latency to approach. Ravens approached the new site significantly slower than the old site (estimate −0.68 ± 0.26 SE, *P* = 0.012; see Fig. 2a and Table S1). Their approach latency was also strongly correlated to the latency for the boars to eat (estimate 0.001 ± 0.0002 SE, *P* < 0.001; see Fig. 2b and Table S1). Visual inspection of the data suggests that this correlation was largely driven by the first 2 d of the experiment in both years, during which both ravens and boars were slower to approach the new site. Because this happened in both years and a similar, albeit weaker, trend was observed in the remaining data, we consider this finding to be robust.

### The total number of ravens was between 6 and 135 ravens, with an average of 71 ravens

**Figure 3:**
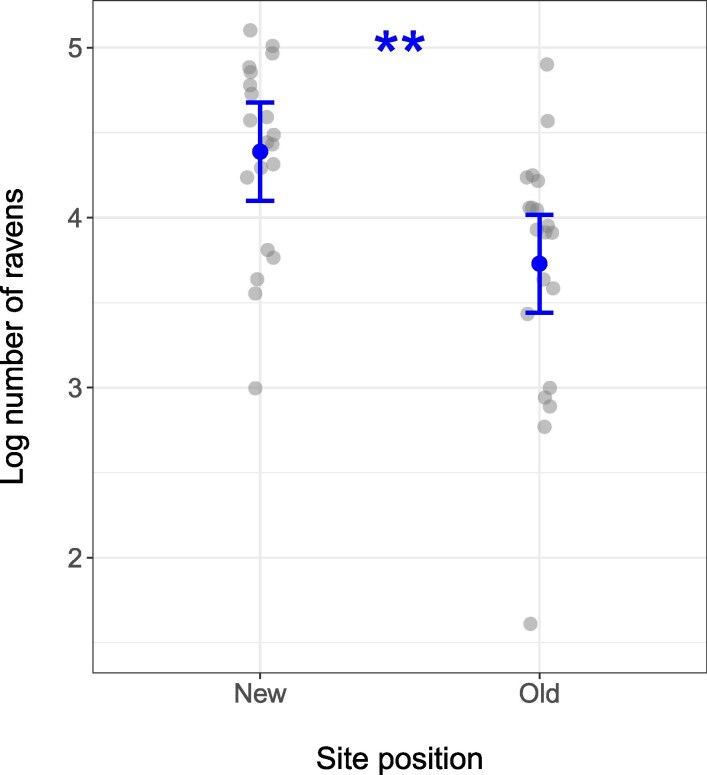
The effect of site on the number of ravens during the 2-sites experiment. The dots represent individual data points. The blue error bars with the dots in the middle respectively represent the lower and upper bounds (95% confidence interval) and mean model fit of the GLM. Detailed effect sizes can be found in Table 2.

**Figure 4:**
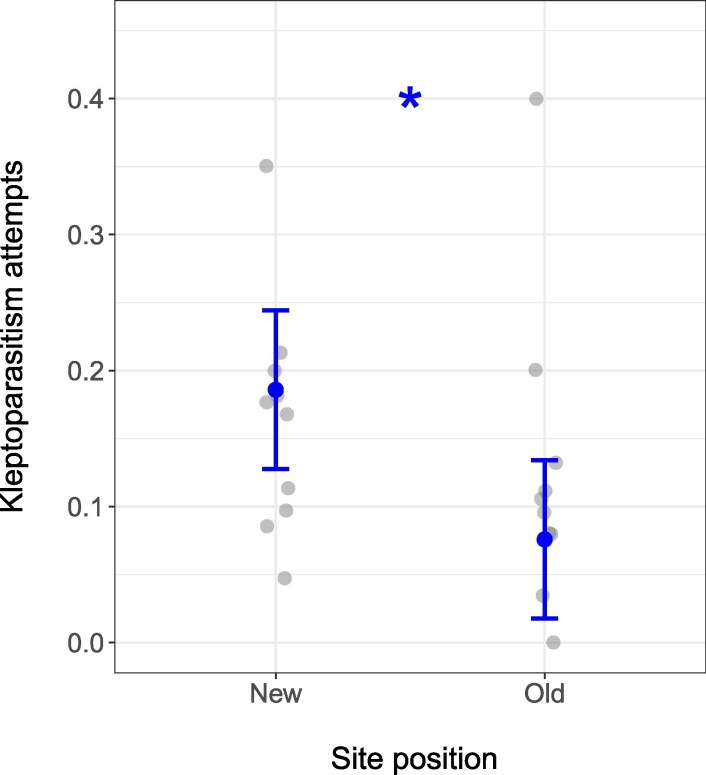
The effect of site on the mean number of kleptoparasitism attempts per raven during the 2-sites experiment. The dots represent individual data points. The blue error bars with the dots in the middle respectively represent the lower and upper bounds (95% confidence interval) and mean model fit of the LM. Detailed effect sizes can be found in Table 2.

**Figure 5:**
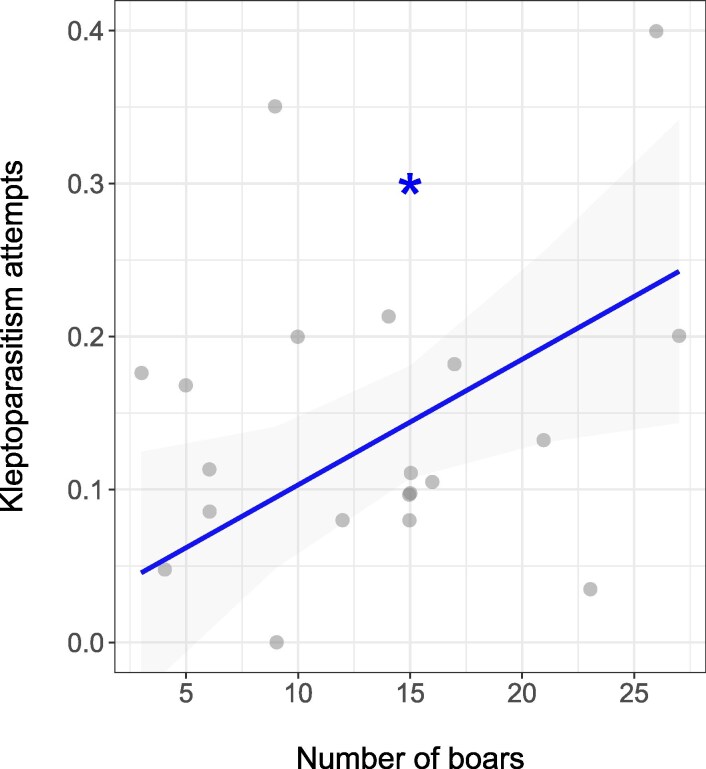
The effect of the number of boars on the ravens' kleptoparasitism attempts during the 2-sites experiment. The dots represent individual data points. The ribbon represents the lower and upper bounds (95% confidence interval) of our LM, with the line as the mean model fit. Detailed effect sizes can be found in Table 2.

Test condition (site), but not time in the experiment, had a significant effect on the ravens' use of the foraging sites. Over the study duration, more ravens foraged at the new site compared with the old site (estimate −0.66 ± 0.20 SE, *P* < 0.002; see Fig. 3 and Table 2), and this difference remained constant throughout the experiment (estimate 0.04 ± 0.04 SE, *P* = 0.249; see Table 2). The number of kleptoparasitism attempts per ravens was between 0 and 0.350, with an average of 0.104 attempts per raven. Test condition (site) and number of boars, had a significant effect on kleptoparasitism attempts. They kleptoparasitized conspecifics more often at the new site compared with the old site (estimate −0.11 ± 0.04 SE, *P* = 0.023; see Fig. 4 and Table 2), and the number of boars had a positive effect on the number of kleptoparasitism attempts toward conspecifics (estimate 0.01 ± 0.002 SE, *P* = 0.021; see Fig. 5 and Table 2). We did not find any effect on the boars manipulations nor the agonistic interactions (see Tables 2 and 3).

**Table 2:** LM regression coefficients for the 2-sites experiment for the log-latency for 5 ravens to approach, for the number of ravens, the mean number of kleptoparasitism attempts, for the mean number of agonistic interactions, and for the mean number of boar manipulations.

Selected models (no model averaging needed)					
	Estimates	SE	CI	*t*	*P*
Two sites experiment					
Log-latency to approach for 5 ravens					
Intercept	4.30	0.34	3.61 to 4.99	12.68	<0.001
Site [Old]	−0.68	0.26	−1.20 to −0.16	−2.67	0.012
Day	−0.03	0.05	−0.13 to 0.06	−0.65	0.517
Latency for boars to eat	0.00	0.00	0.00 to 0.00	4.95	<0.001
*N* = 38; *R*^2^ = 0.617/0.583					
Number of ravens					
Intercept	4.61	0.24	4.13 to 5.10	19.27	<0.001
Site [Old]	−0.66	0.20	−1.07 to −0.25	−3.28	0.002
Day	−0.04	0.04	−0.11 to 0.03	−1.17	0.249
*N* = 40; *R*^2^ = 0.274/0.206					
Mean number of kleptoparasitism attempts per raven					
Intercept	0.06	0.05	−0.06 to 0.17	1.06	0.304
Site [Old]	−0.11	0.04	−0.20 to −0.02	−2.50	0.023
Day	0.00	0.01	−0.01 to 0.02	0.64	0.533
Sum of boars	0.01	0.00	0.00 to 0.02	2.56	0.021
*N* = 20; *R*^2^ = 0.327/0.201					
Mean number of defensive calls per raven					
Intercept	0.14	0.08	−0.02 to 0.30	1.86	0.080
Day	0.00	0.01	−0.03 to 0.03	0.01	0.993
*N* = 20; *R*^2^ = 0.000/−0.056					
Mean number of boar manipulations per ravens					
Intercept	0.17	0.05	0.08 to 0.27	3.80	0.001
Day	−0.01	0.01	−0.02 to 0.01	−0.82	0.423
*N* = 20; *R*^2^ = 0.036/−0.017					
Object experiment					
Log-latency to approach for 5 ravens					
Intercept	5.09	0.83	3.34 to 6.83	6.13	<0.001
Day	−0.03	0.13	−0.32 to 0.25	−0.26	0.798
*N* = 20; *R*^2^ = 0.038/−0.142					
Number of ravens					
Intercept	3.68	0.52	2.55 to 4.81	7.03	<0.001
Object	−0.40	0.35	−1.16 to 0.35	−1.15	0.269
Site [Old]	−0.91	0.36	−1.68 to −0.15	−2.57	0.023
Day	−0.02	0.05	−0.13 to 0.08	−0.47	0.646
Number of boars	0.08	0.04	0.00 to 0.16	2.28	0.040
Object:Site	−2.18	0.54	−3.36 to −1.01	−4.02	0.001
*N* = 20; *R*^2^ Nagelkerke = 0.876					
Mean number of kleptoparasitism attempts per raven					
Intercept	0.08	0.04	−0.01 to 0.17	1.94	0.070
Day	0.08	0.04	−0.01 to 0.17	1.94	0.070
Number of boars	−0.00	0.01	−0.02 to 0.01	−0.10	0.919
*N* = 18; *R*^2^ = 0.001/−0.062					

LM regression coefficients for the object experiment for the log-latency for 5 ravens to approach, for the number of ravens, and for the mean number of kleptoparasitism attempts.

**Table 3:** Full model-average of 3 selected models for the mean number of defensive calls per raven and the mean number of boar manipulations per raven during the object experiment.

Selected models (with model averaging)						
Object experiment						
	Estimates	SE	Adjusted SE	CI	*Z* values	*P*
Mean number of defensive calls per raven						
Intercept	0.13	0.05	0.06	0.02 to 0.25	2.27	0.023
Object	−0.07	0.04	0.04	−0.14 to 0.01	1.71	0.087
Day	−0.01	0.01	0.01	−0.02 to 0.01	1.35	0.177
Number of boars	−0.01	0.01	0.01	−0.02 to 0.01	0.41	0.682
Mean number of boar manipulations per raven						
Intercept	0.01	0.01	0.01	−0.01 to 0.02	0.39	0.695
Day	0.01	0.01	0.01	−0.001 to 0.004	0.88	0.379
Object	−0.01	0.01	0.01	−0.02 to 0.001	0.78	0.434
Site	−0.01	0.01	0.01	−0.02 to 0.01	0.36	0.716

### Object experiment

We did not find a significant effect of time in the experiment on the ravens' latency to approach the food (estimates −0.03 ± 0.13 SE, *P* = 0.798); see Table 2.

#### The total number of ravens was between 0 and 187 ravens, with an average of 59 ravens.

**Figure 6:**
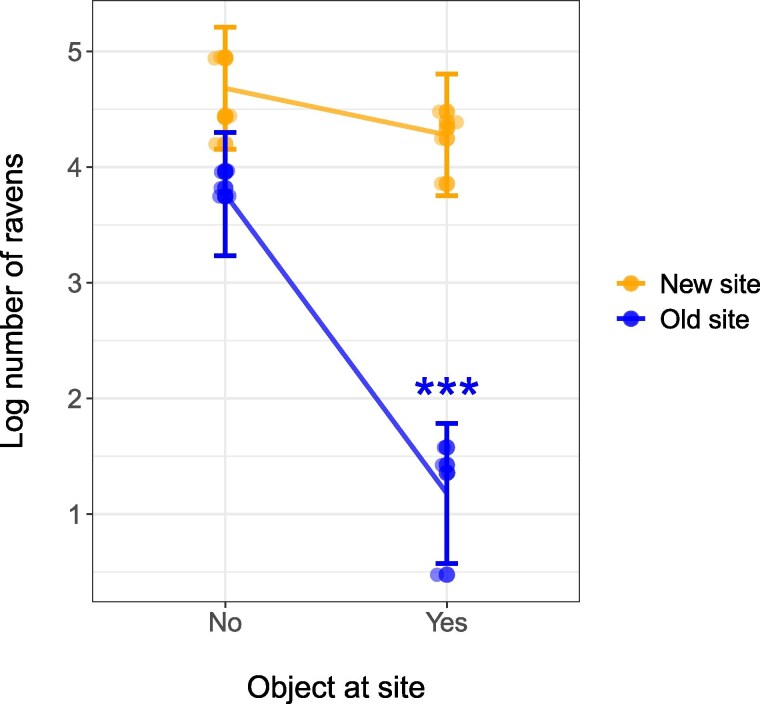
The interactive effects of the object position and the site position. The dots represent the individual data points. The error bars the lower and upper bounds (95% confidence interval) of our GLM and the lines between the error bars the mean model fit. Detailed effect sizes can be found in Table 2.

Our model revealed a significant effect of site and number of boars on the number of ravens, together with a significant effect of the interaction of site (old/new) and presence of the object (estimates: site = −0.91 ± 0.36, number of boars = 0.08 ± 0.04, object:site = −2.18 ± 0.54; *P*-values: site = 0.023, number of boars = 0.040, object:site = 0.001; see Table 2): fewer ravens foraged at the old site when it was paired with an object, while we saw no significant effect of the object at the new site (see Fig. 6 and Table 2). We did not find any effect on kleptoparasitism attempts, agonistic interactions, nor boars manipulations (see Tables 2 and 3).

## Discussion

We here experimentally demonstrate that ravens perceive unfamiliar and unpredictable foraging scenarios as risky and adjust their behavior accordingly by adapting their scrounging strategies to take advantage of the behavior of con- and heterospecifics. In the 2-sites experiment, ravens were slower to approach a unfamiliar feeding site and kleptoparasited conspecifics more there. Their latency to approach was strongly correlated with the behavior of wild boars, indicating that ravens may use heterospecifics as cues to assess safety. Once at the new site, ravens gathered in higher numbers and showed higher rates of kleptoparasitism toward conspecifics than at the familiar site. In the object experiment, while the presence of a familiar but unpredictable object alone did not significantly alter approach latencies, it did reduce foraging activity when placed at the familiar site, suggesting that unpredictability in familiar contexts can trigger avoidance. Together, these findings highlight the ravens' behavioral flexibility and reliance on con- and heterospecifics to navigate changing and uncertain environments. More broadly, our results fit growing comparative evidence that animals can use social information even outside cohesive social groups (Webster 2023), and that mixed-species cues can help individuals respond to both food-related opportunities and predator-related uncertainty (Mukherjee and Bhat 2025).

In the 2-sites experiment, our results on the ravens' longer latency to approach an unfamiliar foraging site indicate that they perceive it as riskier than a familiar one. This is in line with our hypothesis and is supported by extensive literature that uses latency to approach new resources, objects, or areas as a measure for neophobia (eg Mettke-Hofmann et al. 2009; Audet et al. 2016; Miller et al. 2022). Moreover, we found that ravens directed more kleptoparasitism attempts toward conspecifics at the new foraging site compared with the familiar one, and that the number of kleptoparasitism attempts had a positive correlation with the number of boars present at the feeding site. Intraspecific kleptoparasitism is an opportunistic foraging tactic regularly observed in (some) ravens (Gallego-Abenza et al. 2020). The predicted increase in kleptoparasitism at the unfamiliar site lends experimental support to the idea that individuals flexibly adjust their social foraging strategies in response to perceived risk. By exploiting the foraging success of conspecifics, individuals reduce their own exposure to potentially dangerous environments. Our results also align with a previous correlational study comparing ravens foraging strategies under varying levels of perceived predation risk. Specifically, ravens showed higher kleptoparasitism attempts toward conspecifics in the presence of potential predators (wolves) compared with non-predators (wild boars) (Bugnyar and Kotrschal 2002). An alternative but not mutually exclusive interpretation could be that ravens use kleptoparasitism as a strategy to deal with increased competition from heterospecifics. This highlights the complex relation between scavenging ravens and the species they co-feed with, which is further supported by the fact that while they compete with them, ravens only approached the sites after the boars did, possibly using them to determine the safety of the foraging area. This pattern is consistent with work outside scavenger systems showing that heterospecific and mixed-species cues can inform both predator avoidance and foraging decisions, suggesting that the ravens' use of boars may reflect a general mechanism of cross-species information use under uncertainty (Webster 2023; Mukherjee and Bhat 2025). Future studies should explore this complex relationship in a completely wild context. Contrary to our expectation, ravens did not engage more in agonistic interactions at the new site, suggesting that levels of kleptoparasitism were not directly linked to rates of aggression.

We also found that more ravens visited the unfamiliar foraging site when compared with the familiar site. Intuitively, this result seems to contradict the idea that the new site is perceived as riskier. However, large groups often provide safety in numbers through mechanisms such as the dilution effect, which reduces the likelihood that any individual will be targeted by a predator, and the many-eyes effect, which enhances predator detection (Lehtonen and Jaatinen 2016). By aggregating near the unfamiliar site, ravens may benefit from reduced individual risk, consistent with strategies where forming large groups decreases predation risk in the foraging context (Hamilton 1971; Morse 1977). One might argue that, once ravens had found out that there is little risk at the new site, their increased numbers at the new site could indicate their interest to explore this site. However, the consistency over time in the behavioral pattern indicative for neophobia (see below) speak against this interpretation.

Contrary to our prediction, the latency to approach, the number of ravens and the number of kleptoparasitic attempts remained higher at the new site than the old site for the entire experiment. These findings indicate that the ravens kept perceiving the new foraging site as relatively unfamiliar throughout the study period of 1 mo per year. It is thus likely that the duration of our experiments was not long enough for the ravens to fully familiarize with the new site. Moreover, the new site was only spatially but not temporally predictable, which might have delayed any habituation. Possibly, the low levels of agonistic interactions observed between ravens reflected the low level of familiarization. Note also that our study focused on group-level analysis. It might well be that on the individual level, some ravens were prone to exploit the new site either alone or together with conspecifics, whereas others tended to stay at the old site. It is thus possible that the distribution of the ravens between the 2 sites was affected by individual differences connected, for example, to personality (Réale et al. 2007) or age (Weißenborn et al. 2025), as both factors have been shown to impact neophilia and neophobia in ravens (Heinrich 1995; Miller et al. 2015). Future studies should explore the behavior of individual ravens in similar foraging scenarios to shed a light on those factors.

In the object experiment, the results support our hypothesis that introducing a familiar but unpredictable object next to a feeding site alters its perceived risk for ravens. However, the aversive effect on foraging activity appears to be strongest at the familiar site, where raven visitations dropped to low numbers when the object was present, compared with both the new site and control conditions. This pattern suggests that ravens are highly susceptible to changes in the immediate environment, especially so when the change is violating their expectations at a familiar location. Conversely, when foraging at the new site, ravens appeared more tolerant to the presence of the bike, possibly because having less expectations about an environment allows higher tolerance to change within it. This context-dependent risk sensitivity aligns with findings in other scavengers and wildlife species. Similar results were found, for example, in a study by Harris and Knowlton 2001, where coyotes had stronger neophobic reactions in familiar environments compared with unfamiliar ones. Similar behavioral flexibility has been observed in corvids, including ravens, which adapt their foraging and risk-taking strategies depending on previous experience and local environmental stability (Marzluff and Angell 2005). Our findings therefore support the idea that animals may respond not only to unfamiliarity itself, but to the interaction between unfamiliarity and prior expectations about a place.

As a final note, we would like to point out that the value in unfamiliarity differs between our 2 experiments. While the new site represented an additional location to access food, the unpredictable object itself had no such value. This inherent difference in unfamiliarity may partially explain why there is such a strong neophobic response with the novel object, unlike with the novel foraging site, and supplement our explanation concerning violation of expectation. Previous studies on corvids revealed that these birds respond differently to different types of novelty (eg Vernouillet and Kelly 2020; Miller et al. 2022). Yet, it might be possible that on the individual level, birds are consistent in their choices. Future studies could focus on the foraging behavior of individual ravens: we would expect birds that are more prone to taking risks (ie bolder and/or more exploratory) to prefer to forage at the new site and be less affected by unpredictable events like objects than birds that are risk-averse (ie shyer and/or less exploratory) and prefer to utilize the old site.

In both experiments, we found evidence that the initial approach to either site depended on the behavior of wild boars. Ravens appear to use the boars as initiators or “demonstrators,” waiting for them to approach a foraging site before doing so themselves. This happened independently from the familiarity and predictability of the foraging environment. A possible explanation is that ravens, as scavengers, are used to eating together with, and stealing from, heterospecifics; hence, the absence of boars at a familiar site could have alerted them to a possible danger, while them foraging at a risky site could be taken as an indication that it was safe. This interpretation fits broader comparative work suggesting that shared resource sites can function as hubs of multispecies interaction, where animals are exposed simultaneously to opportunities, competitors, and social cues from other species (Webster 2023; Griffiths et al. 2025). In that sense, the boars may have served not only as competitors or targets of scrounging, but also as an important component of the ravens' information landscape. Interestingly, we did not find that ravens manipulated wild boars more often in the risky than the familiar condition. These results suggest that scrounging attempts directed toward heterospecifics were less prominent than those toward conspecifics.

## Conclusion

Altogether, our findings indicate that generalist scavengers like ravens can take advantage of changing environments not only because of their broad diet, but also because they are able to use con- and heterospecifics as sources of information and means to access food. Many of the ravens' responses to novelty and unpredictability in our experiments were in the form of changes in behavior toward conspecifics, underlining the importance of social context during foraging. Moreover, while some species may perceive the presence of heterospecific competitors as an additional challenge, ravens appear to leverage their behavior to mitigate environmental risk. These capacities reflect their well-known socio-cognitive flexibility and likely contribute to their success in different environments, including those characterized by frequent change (Jolles et al. 2013; Orr et al. 2019). Such flexibility may be particularly beneficial in human-disturbed systems, where disturbance can alter scavenger assemblages and carrion-removal dynamics while still favoring behaviorally flexible generalists (Bartel et al. 2024; Allen et al. 2026).

## Supplementary Material

arag093_Supplementary_Data

## Data Availability

Analyses reported in this article can be reproduced using the data provided by Damini et al. (2026).
