# Supplementary material for: Ravens (Corvus corax) use conspecifics and heterospecifics to navigate risky foraging scenarios
Source: Behav Ecol. 2026 Jul 30;37(5):arag093. doi: 10.1093/beheco/arag093 (PMC13475124; doi:10.1093/beheco/arag093)
Supplement: Supplementary file 1 [file arag093_supplementary_data.zip › Revised4_Clean_Anonymous_Supplementary_Material.docx]

# **Supplementary material**

## Raven information

### Table S1: List of marked ravens that participated in our experiments, including their name (Name), in which year they participated in the experiment (Observed in), their sex as determined through blood test (Sex), the exact year they hatched or an approximation based on physical characteristics (Hatching year), and whether they were born in the wild or in captivity (Origin).

| Name | Observed in | Sex | Hatching year | Origin |
| --- | --- | --- | --- | --- |
| Auguste | 2022, 2023 | F | 2021 | Captive bred |
| Bamboo | 2023 | F | 2019 | Wild caught |
| Cigar | 2023 | F | <2020 | Wild caught |
| Cheyenne | 2022, 2023 | F | 2015 | Wild caught |
| Chowder | 2023 | M | 2022 | Captive bred |
| Clove | 2022, 2023 | M | 2017 | Captive bred |
| Cobain | 2022 | M | 2021 | Captive bred |
| Cotton | 2023 | F | <2020 | Wild caught |
| Dandelion | 2022, 2023 | M | 2017 | Captive bred |
| Echo | 2022, 2023 | M | 2019 | Wild caught |
| Eragon | 2022 | M | 2020 | Captive bred |
| Flo | 2023 | F | 2022 | Wild caught |
| Foxtrott | 2022, 2023 | M | 2021 | Captive bred |
| Garance | 2022 | M | 2021 | Captive bred |
| Ginza | 2022 | M | 2020 | Captive bred |
| Gisla | 2022, 2023 | F | 2016 | Captive bred |
| Gwen | 2022, 2023 | F | 2018 | Wild caught |
| Hippolyte | 2022 | M | 2021 | Captive bred |
| Ikea | 2023 | F | <2020 | Wild caught |
| James Bond | 2023 | F | >2020 | Wild caught |
| Kibi | 2022, 2023 | M | 2019 | Wild caught |
| Lexi | 2023 | F | <2020 | Wild caught |
| Mayer | 2022 | M | 2017 | Wild caught |
| Mombu | 2022 | M | 2018 | Wild caught |
| Navy | 2023 | F | 2022 | Captive bred |
| Padma | 2023 | F | <2019 | Wild caught |
| Raikiri | 2023 | M | 2020 | Wild caught |
| Rollo | 2022 | M | 2016 | Captive bred |
| Salsa | 2022 | M | 2021 | Captive bred |
| T-Rex | 2022, 2023 | M | <2015 | Wild caught |
| Tweety | 2022 | F | 2018 | Wild caught |
| Ueno | 2022, 2023 | M | 2020 | Captive bred |
| Unesco | 2023 | F | 2019 | Wild caught |
| Xmas | 2023 | F | <2020 | Wild caught |
| Yama | 2023 | F | 2020 | Wild caught |
| Zwiebel | 2023 | F | <2022 | Wild caught |

## Two-sites experiment

### Table S2: Latency to approach

*Model selection table for the log-latency for a minimum of five ravens to approach during the two-sites experiment. With ‘+’ indicating an additive term, and ‘*’ both an interaction and an additive term. Model 3 was selected.*

|  | Model selection - Log-latency to approach for 5 ravens | | | | | | |
| --- | --- | --- | --- | --- | --- | --- | --- |
| Models | Variables | K | AICc | Delta AICc | AICc Wt | Cum. Wt | LL |
| Model 3 | site+ day +latency boars | 5 | 92.02 | **0.00** | 0.79 | 12. 0.79 | **-40.07** |
| Model 2 | site* day +latency boars | 6 | 94.69 | 2.67 | 0.21 | 1.00 | **-39.99** |
| Model 5 | site+day | 4 | 109.97 | 17.95 | 0.00 | 1.00 | -50.38 |
| Model 4 | site* day | 5 | 110.70 | 18.68 | 0.00 | 1.00 | **-49.41** |
| Model 1 | day | 3 | 119.87 | 27.85 | 0.00 | 1.00 | **-56.58** |

### Table S3: Number of ravens

Model *selection* table for the number of ravens during the two-sites experiment. With ‘+’ indicating an additive term, and ‘*’ both an interaction and an additive term. Model 5 was selected.

| Model selection - Number of ravens | | | | | | | |
| --- | --- | --- | --- | --- | --- | --- | --- |
| Models | Variables | K | AICc | Delta AICc | AICc Wt | Cum. Wt | LL |
| Model 5 | site + day | 4 | 83.35 | **0.00** | 0.61 | 0.61 | **-37.10** |
| Model 4 | site*day | 5 | 85.94 | 2.59 | 0.17 | 0.78 | **-37.10** |
| Model 3 | site+ day+ latency boars | 5 | 85.96 | 2.61 | 0.17 | 0.95 | -37.10 |
| Model 2 | site *day+ latency boars | 6 | 88.72 | 5.37 | 0.04 | 0.99 | **-37.10** |
| Model 1 | day | 3 | 91.06 | 7.71 | 0.01 | 1.00 | **-42.20** |

### Table S4: Scrounging

*Model selection table for the mean number of kleptoparasitism attempts per raven during the two-sites experiment. With ‘+’ indicating an additive term, and ‘*’ both an interaction and an additive term. Model 3 was selected.*

| Model selection - Mean number of kleptoparasitism attempts per raven | | | | | | | |
| --- | --- | --- | --- | --- | --- | --- | --- |
| Models | Variables | K | AICc | Delta AICc | AICc Wt | Cum. Wt | LL |
| Model 3 | site+ day+ latency boars | 5 | -37.58 | **0.00** | 0.48 | 0.48 | **25.93** |
| Model 1 | day | 3 | -36.48 | 2.10 | 0.28 | 0.76 | **21.99** |
| Model 2 | site*day+ latency boars | 6 | -34.50 | 3.08 | 0.10 | 0.86 | 26.48 |
| Model 5 | site+day | 4 | -34.35 | 3.23 | 0.10 | 0.96 | **22.51** |
| Model 4 | site*day | 5 | -32.79 | 4.79 | 0.04 | 1.00 | **23.54** |

### Table S5: Number of agonistic interactions

*Model selection table for the mean number of agonistic interactions per raven during the two-sites experiment. With ‘+’ indicating an additive term, and ‘*’ both an interaction and an additive term. Model 1 was selected.*

| Model selection - Mean number of defensive calls per raven | | | | | | | |
| --- | --- | --- | --- | --- | --- | --- | --- |
| Models | Variables | K | AICc | Delta AICc | AICc Wt | Cum. Wt | LL |
| Model 1 | day | 3 | -12.24 | **0.00** | 0.71 | 0.71 | **9.87** |
| Model 5 | site+day | 4 | -9.50 | 2.74 | 0.18 | 0.89 | **10.08** |
| Model 3 | site*day | 5 | -6.00 | 6.24 | 0.03 | 0.96 | **10.14** |
| Model 4 | site+day+ number of boars | 5 | -5.89 | 6.35 | 0.03 | 0.99 | 10.09 |
| Model 2 | site*day+ number of boars | 6 | -1.82 | 10.41 | 0.00 | 1.00 | 10.14 |

### Table S6: Number of boar manipulation

*Model selection table for the mean number of boar manipulations per raven during the two-sites experiment. With ‘+’ indicating an additive term, and ‘*’ both an interaction and an additive term. Model 1 was selected.*

| Model selection - Mean number of boar manipulations per ravens | | | | | | | |
| --- | --- | --- | --- | --- | --- | --- | --- |
| Models | Variables | K | AICc | Delta AICc | AICc Wt | Cum. Wt | LL |
| Model 1 | day | 3 | -32.53 | **0.00** | 0.69 | 0.69 | **20.01** |
| Model 5 | site+day | 4 | -29.62 | 2.90 | 0.16 | 0.85 | **20.14** |
| Model 3 | site*day | 5 | -28.88 | 3.65 | 0.11 | 0.96 | **21.58** |
| Model 4 | site+day+ number of boars | 5 | -26.15 | 6.37 | 0.03 | 0.99 | 20.22 |
| Model 2 | site*day+ number of boars | 6 | -24.70 | 7.82 | 0.01 | 1.00 | 21.58 |

Object experiment

### Table S7: Latency to approach

*Model selection table for the log-latency to approach during the object experiment. With ‘+’ indicating an additive term, and ‘*’ both an interaction and an additive term. Model 1 was selected.*

| Model selection - Log-latency to approach for 5 ravens | | | | | | | |
| --- | --- | --- | --- | --- | --- | --- | --- |
| Models | Variables | K | AICc | Delta AICc | AICc Wt | Cum. Wt | LL |
| Model 1 | day | 3 | 83.79 | **0.00** | 0.55 | 0.55 | **-38.15** |
| Model 9 | site+day | 4 | 86.59 | 2.80 | 0.14 | 0.69 | **-37.96** |
| Model 7 | object+day | 4 | 86.61 | 2.81 | 0.14 | 0.83 | **-37.97** |
| Model 8 | day+latency boars | 4 | 86.95 | 3.16 | 0.11 | 0.94 | -38.14 |
| Model 5 | object+site+day | 5 | 89.85 | 6.06 | 0.03 | 0.97 | -37.78 |
| Model 6 | object+day+latency boars | 5 | 90.23 | 6.43 | 0.02 | 0.99 | -37.97 |
| Model 4 | object+site+day+latency boars | 6 | 92.99 | 9.20 | 0.01 | 1.00 | -37.27 |
| Model 3 | object*site+day | 6 | 93.94 | 10.15 | 0.00 | 1.00 | **-37.74** |
| Model 2 | object*site+day+latency boars | 7 | 97.68 | 13.89 | 0.00 | 1.00 | **-37.17** |

### Table S8: Number of ravens

a) Model selection table for the number of ravens during the object experiment. With ‘+’ indicating an additive term, and ‘*’ both an interaction and an additive term. Model 2 was selected.

Model selection - Number of ravens

| Models | Variables | K | AICc | Delta AICc | AICc Wt | Cum. Wt | LL |
| --- | --- | --- | --- | --- | --- | --- | --- |
| Model 2 | object*site+day+number of boars | 7 | 46.93 | **0.00** | 0.82 | 0.82 | **-11.37** |
| Model 3 | object*site+day | 6 | 50.07 | 3.14 | 0.17 | 0.99 | **-15.80** |
| Model 4 | object+site+day+number of boars | 6 | 57.10 | 10.17 | 0.01 | 1.00 | **-19.05** |
| Model 5 | object+site+day | 5 | 58.05 | 11.12 | 0.00 | 1.00 | -21.88 |
| Model 9 | site+day | 4 | 67.64 | 20.71 | 0.00 | 1.00 | -28.49 |
| Model 7 | object+day | 4 | 73.32 | 26.39 | 0.00 | 1.00 | -31.33 |
| Model 6 | object+day+number of boars | 5 | 73.85 | 26.93 | 0.00 | 1.00 | -29.62 |
| Model 8 | day+number of boars | 4 | 75.18 | 28.25 | 0.00 | 1.00 | **-32.16** |
| Model 1 | day | 3 | 76.36 | 29.43 | 0.00 | 1.00 | **-34.43** |

b) Number of ravens post hoc test results. "No object" and "Yes object" indicate the presence of the object at a given site. "New" and "Old" the position of the site.

| Contrasts |  | Estimate | | SE | df | t-ratio | p |
| --- | --- | --- | --- | --- | --- | --- | --- |
| No object, New | – Yes object, New | | 0.40 | 0.35 | 13 | 1.15 | 0.664 |
| No object, New | – No object, Old | | 0.91 | 0.36 | 13 | 2.57 | 0.095 |
| No object, New | – Yes object, Old | | 3.50 | 0.36 | 13 | 9.61 | **<0.001** |
| Yes object, New | – No object, Old | | 0.51 | 0.34 | 13 | 1.51 | 0.462 |
| Yes object, New | – Yes object, Old | | 3.10 | 0.38 | 13 | 8.09 | **<0.001** |
| No object, Old | – Yes object, Old | | 2.59 | 0.38 | 13 | 6.74 | **<0.001** |

### Table S9: Kleptoparasitism

Model selection table for the number of kleptoparasitism attempts per raven during the object experiment. With ‘+’ indicating an additive term, and ‘*’ both an interaction and additive term. Model 1 was selected

| Model selection - Mean number of kleptoparasitism attempts per raven | | | | | | | | |
| --- | --- | --- | --- | --- | --- | --- | --- | --- |
| Models | Variables | K | AICc | Delta AICc | AICc Wt | Cum. Wt | LL |  |
| Model 1 | day | 3 | -35.28 | **0.00** | 0.56 | 0.56 | **21.50** |  |
| Model 3 | object+site+day+number of boars | 6 | -33.00 | 2.28 | 0.18 | 0.74 | **26.32** |  |
| Model 5 | object+day | 4 | -32.33 | 2.95 | 0.13 | 0.86 | **21.70** |  |
| Model 4 | object+site+day | 5 | -31.71 | 3.57 | 0.09 | 0.96 | 23.36 |  |
| Model 2 | object*site+day+number of boars | 7 | -30.15 | 5.13 | 0.04 | 1.00 | 27.67 |  |

### Table S10: Number of agonistic interactions

Model selection table for the number of defensive calls per raven during the object experiment. With ‘+’ indicating an additive term, and ‘*’ both an interaction and an additive term. Models 7, 1, and 5 were selected.

| Model selection - Mean number of defensive calls per raven | | | | | | | |
| --- | --- | --- | --- | --- | --- | --- | --- |
| Models | Variables | K | AICc | Delta AICc | AICc Wt | Cum. Wt | LL |
| Model 7 | site+day | 4 | -34.56 | **0.00** | 0.35 | 0.35 | **22.82** |
| Model 1 | day | 3 | -34.07 | **0.49** | 0.28 | 0.63 | **20.89** |
| Model 5 | object+site+day | 5 | -33.92 | **0.64** | 0.26 | 0.89 | **24.46** |
| Model 6 | object+day+number of boars | 5 | -30.89 | 3.67 | 0.06 | 0.94 | 22.95 |
| Model 4 | site*day+ number of boars | 6 | -29.49 | 5.07 | 0.03 | 0.97 | 24.56 |
| Model 3 | object*site+day | 6 | -29.32 | 5.24 | 0.03 | 1.00 | 24.48 |
| Model 2 | object*site+day+number of boars | 7 | -23.96 | 10.60 | 0.00 | 1.00 | 24.58 |

### Table S11: Number of boar manipulation

*Model selection table for the number of boar manipulations per raven during the object experiment. With ‘+’ indicating an additive term, and ‘*’ both an interaction and an additive term. Models 1, 7, and 5 were selected.*

| Model selection - Mean number of boar manipulation per ravens | | | | | | | |
| --- | --- | --- | --- | --- | --- | --- | --- |
| Models | Variables | K | AICc | Delta AICc | AICc Wt | Cum. Wt | LL |
| Model 1 | day | 3 | -96.36 | **0.00** | 0.37 | 0.37 | **52.04** |
| Model 7 | object+day | 4 | -95.86 | **0.50** | 0.29 | 0.29 | **53.47** |
| Model 5 | object+site+day | 5 | -94.50 | **1.86** | 0.15 | 0.15 | **54.75** |
| Model 6 | object+day+number of boars | 5 | -93.66 | 2.70 | 0.10 | 0.10 | 54.33 |
| Model 3 | object*site+day | 6 | -93.03 | 3.33 | 0.07 | 0.07 | 56.33 |
| Model 4 | object+site+day+number of boars | 6 | -90.28 | 6.08 | 0.02 | 0.02 | 54.96 |
| Model 2 | object*site+day+number of boars | 7 | -88.03 | 8.33 | 0.01 | 0.01 | 56.61 |

# Appendix 1: Schedule for the experiments

*The schedules showing on which days which setup occurred.*

**2022 two-sites experiment**

| SUN | MON | TUE | WED | THU | FRI | SAT |
| --- | --- | --- | --- | --- | --- | --- |
| 15-05 | 16-05 | 17-06 | 18-05 | 19-05 | 20-05 | 21-05 |
|  | 2-sites |  |  | 2-sites |  |  |
| 22-05 | 23-05 | 24-05 | 25-05 | 26-05 | 27-05 | 28-05 |
|  |  |  | 2-sites |  | 2-sites |  |
| 29-05 | 30-05 | 31-05 | 01-06 | 02-06 | 03-06 | 04-06 |
|  | 2-sites |  |  | 2-sites |  |  |
| 04-06 | 05-06 | 06-06 | 07-06 | 08-06 | 09-06 | 10-06 |
|  |  |  | 2-sites |  | 2-sites |  |
| 11-06 | 12-06 | 13-06 | 14-06 | 15-06 | 16-06 | 17-06 |
|  |  |  |  |  |  |  |
| 18-06 | 19-06 | 20-06 | 21-06 | 22-06 | 23-06 | 24-06 |
|  |  | 2-sites |  | 2-sites |  |  |

**2023 two-sites experiment**

| SUN | MON | TUE | WED | THU | FRI | SAT |
| --- | --- | --- | --- | --- | --- | --- |
| 04-02 | 04-03 | 04-04 | 04-05 | 04-06 | 04-07 | 04-08 |
|  |  |  |  |  |  |  |
| 04-09 | 04-10 | 04-11 | 04-12 | 04-13 | 04-14 | 04-15 |
|  |  |  |  |  |  |  |
| 04-16 | 04-17 | 04-18 | 19-04 | 04-20 | 21-04 | 04-22 |
|  |  |  | 2-sites |  | 2-sites |  |
| 04-23 | 04-24 | 25-04 | 04-26 | 27-04 | 04-28 | 04-29 |
|  |  | 2-sites |  | 2-sites |  |  |
| 30-04 | 05-01 | 02-05 | 05-03 | 05-04 | 05-05 | 05-06 |
| 2-sites |  | 2-sites |  |  |  |  |
| 05-07 | 08-05 | 05-09 | 05-10 | 11-05 | 05-12 | 05-13 |
|  | 2-sites |  |  | 2-sites |  |  |
| 05-14 | 05-15 | 16-05 | 05-17 | 05-18 | 19-05 | 05-20 |
|  |  | 2-sites |  |  | 2-sites |  |

**2023 object experiment**

| SUN | MON | TUE | WED | THU | FRI | SAT |
| --- | --- | --- | --- | --- | --- | --- |
| 05-28 | 05-29 | 30-05 | 31-05 | 01-06 | 06-02 | 06-03 |
|  |  | object old | object old | object new |  |  |
| 06-04 | 05-06 | 06-06 | 07-06 | 06-08 | 09-06 | 06-10 |
|  | object new |  | object old |  | object old |  |
| 06-11 | 12-06 | 13-06 | 06-14 | 06-15 | 16-06 | 06-17 |
|  | object new | object new |  |  | object old |  |
| 06-18 | 19-06 | 06-20 | 06-21 | 06-22 | 06-23 | 06-24 |
|  | object new |  |  |  |  |  |

Supplementary figure 1: Boar enclosure

View of the boar enclosure from above. The perimeter of the boar enclosure is roughly outlined by the yellow circle, while the red dots show the approximate position of the feeding sites.

Insert Supplementary Figure 1
